# CTCF contributes in a critical way to spermatogenesis and male fertility

**DOI:** 10.1038/srep28355

**Published:** 2016-06-27

**Authors:** Abrahan Hernández-Hernández, Ingrid Lilienthal, Nanaho Fukuda, Niels Galjart, Christer Höög

**Affiliations:** 1 Karolinska Institutet, Department of Cell and Molecular Biology, Berzelius väg 35, 171 77 Stockholm, Sweden; 2 Department of Cell Biology and Genetics, Erasmus MC, 2040 CA Rotterdam, The Netherlands

## Abstract

The CCCTC-binding factor (CTCF) is an architectural protein that governs chromatin organization and gene expression in somatic cells. Here, we show that CTCF regulates chromatin compaction necessary for packaging of the paternal genome into mature sperm. Inactivation of *Ctcf* in male germ cells in mice (*Ctcf*-cKO mice) resulted in impaired spermiogenesis and infertility. Residual spermatozoa in *Ctcf*-cKO mice displayed abnormal head morphology, aberrant chromatin compaction, impaired protamine 1 incorporation into chromatin and accelerated histone depletion. Thus, CTCF regulates chromatin organization during spermiogenesis, contributing to the functional organization of mature sperm.

In the gonads of mammalian organisms, diploid germ cells give rise to haploid gametes, egg and sperm. The male germ cell differentiation process, spermatogenesis, takes place in testis^1^,^2^. During spermatogenesis, spermatogonial stem cells act as a source for a continuous production of sperm. Differentiating spermatogonia undergo several rounds of mitotic cell divisions and give rise to spermatocytes, which undergo two meiotic cell divisions, resulting in the formation of round haploid spermatids. The round spermatids subsequently undergo a series of morphological changes, in a process known as spermiogenesis, giving rise to mature sperm (also called spermatozoa)^3^,^4^. An important aspect of spermiogenesis involves the reorganization and compaction of the haploid genome, allowing it to be packaged into the head of spermatozoa^4^. Chromatin compaction during spermiogenesis in mammals is made possible through the replacement of most histones with sperm-specific basic proteins, protamine 1 (PRM1) and protamine 2 (PRM2)^4^,^5^, both proteins being essential for sperm formation^3^,^6^,^7^,^8^. A small fraction of histones (2%) remains bound to chromatin in association with promoter regions and repetitive DNA sequences, in spermatozoa^9^,^10^,^11^. The functional role of histone retention in mature sperm, however, is not known.

CTCF is a highly conserved DNA binding protein that regulates higher order chromatin structure and genome topology in somatic cells, acting as a global regulator of gene transcription^12^,^13^,^14^,^15^. CTCF has a DNA binding motif consisting of eleven zinc finger domains, recognizing tens of thousands DNA sites across the mammalian genome^16^. Binding of CTCF to DNA has been shown to contribute to the formation of three-dimensional DNA loops, mediating long-range chromatin interactions between different regulatory sequences in somatic cells^15^,^17^. Interestingly, in round spermatids CTCF has been shown to be present on promoter regions of genes that are actively transcribed in cancer cell lines^18^, suggesting that CTCF contributes to chromatin organization and gene expression in round spermatids. The functional role of CTCF in male germ cells, however, has not yet been analyzed, as inactivation of *Ctcf* results in embryonic lethality^19^.

We have here studied the role of CTCF during male germ cell development. A male germ line-specific conditional knockout mouse model (*Ctcf-cKO*) was generated, where *Ctcf* was inactivated in pre-leptotene spermatocytes. *Ctcf*-cKO mice displayed seminiferous tubule atrophy, accompanied by low sperm counts and infertility. Elongating spermatids in the testis and mature spermatozoa in the cauda epididymis of *Ctcf*-cKO mice displayed major defects in sperm head formation and chromatin compaction. Genes, expressed in round spermatids in the testis of wild-type mice and contributing to sperm formation, were found to be down-regulated in *Ctcf*-cKO mice. Furthermore, mature spermatozoa in *Ctcf*-cKO mice showed abrogated histone retention and PRM1 recruitment to chromatin. Thus, CTCF contributes in a critical way to the differentiation process that controls formation of mature sperm.

## Results

### Conditional targeting of *Ctcf* in the testis

In order to study the function of CTCF during spermatogenesis, *Ctcf* was conditionally targeted in male germ cells. We crossed mice carrying *Ctcf*-floxed alleles with *Stra8-iCre* transgenic mice^19^,^20^, generating a *Stra8-iCre-Ctcf*^* wt/f*^ heterozygote strain. The Stra8 promoter drives expression of the Cre recombinase in spermatogonia and in pre-leptotene spermatocytes in the testis of male mouse, allowing one to study the functions of conditionally inactivated genes in spermatocytes undergoing meiosis and during spermiogenesis^20^. We found that conditional targeting of the *Ctcf* gene was restricted to the testis in mice heterozygous for the floxed *Ctcf* allele (*Stra8-iCre-Ctcf*^* wt/f*^) (Supplementary Fig. S1). Isolation of different testicular cell populations from *Stra8-iCre-Ctcf*^* wt/f*^ mice using FACS, identified the deleted version of the floxed *Ctcf* allele in primary spermatocytes, secondary spermatocytes and in round haploid cells, showing that the initial Cre targeting events take place in spermatocytes (Supplementary Fig. S1). In agreement with this, *LacZ* gene expression (which is activated upon conditional targeting of a floxed *Ctcf* allele) was observed in primary spermatocytes at the pre-leptotene stage of spermatogenesis (Supplementary Fig. S1).

We thereafter inter-crossed *Stra8-iCre-Ctcf*^* wt/f*^ mice, to simultaneously inactivate both alleles of *Ctcf*, generating a *Ctcf* conditional knockout mouse strain (*Stra8-iCre-Ctcf*^*f*/*Δ*^, hereafter referred to as *Ctcf*-cKO). We compared the levels of CTCF in wild-type and *Ctcf*-cKO littermates, by immunoblotting of testis extracts, and found that *Ctcf*-cKO mice showed a 60% reduction in CTCF protein levels (Supplementary Fig. S1). CTCF has been shown to be expressed in all cell types of the testis^21^, strongly suggesting that the residual expression of CTCF in the testes of *Ctcf*-cKO mice, was due to CTCF expression in testicular cell types not targeted by the *Stra8-iCre* approach used here. In agreement with this, while immunostaining of paraffin-embedded testis sections did not reveal CTCF expression in spermatocytes and spermatids in *Ctcf*-cKO mice (Supplementary Fig. S2), expression of CTCF was observed in Sertoli cells, Leydig cells and spermatogonial cells in the testis of *Ctcf*-cKO mice. Thus, CTCF expression is impaired in spermatocytes and spermatids in the testis of *Ctcf*-cKO mice.

### *Ctcf*-cKO mice display impaired spermiogenesis and infertility

**Figure 1:**
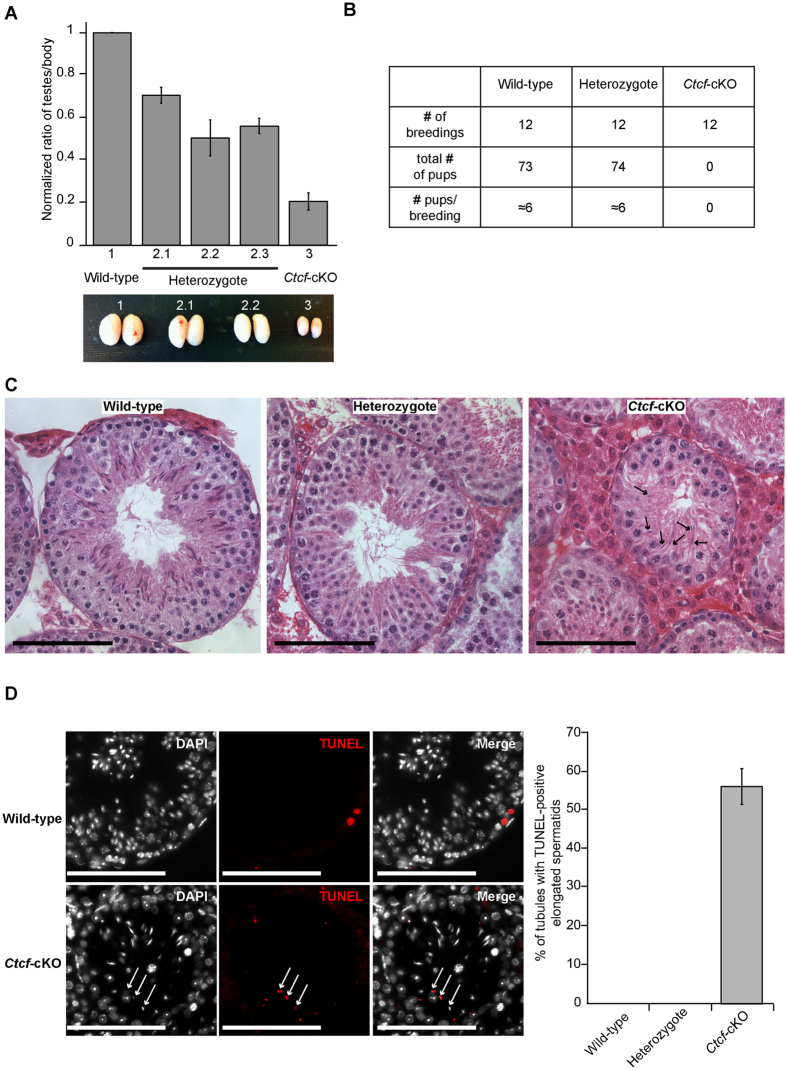
Conditional inactivation of *Ctcf* severely disrupts testis morphology and results in infertility and elongated spermatids apoptosis. (**A**) Testis size was reduced in the absence of *Ctcf*. Testes weight was plotted as a function of body weight in wild-type (1) (n = 7), heterozygote (2.1, 2.2, 2.3) (n = 3 for each genotype), and *Ctcf*-cKO (3) (n = 12) mice. Ratio testes/body were normalized against the wild-type littermates. (**B**) Breeding attempts showed that the *Ctcf*-cKO mice were infertile. (**C**) Hematoxylin and eosine staining of paraffin sections of seminiferous tubules from wild-type, heterozygote and *Ctcf*-cKO mice. Arrows indicate few elongated spermatids found in some of the seminiferous tubules in the Ctcf-*cKO* testis sections. (**D**) Wild-type and *Ctcf*-cKO testis sections were labeled using a TUNEL assay. TUNEL-positive elongated spermatids are indicated by arrows. Right panel shows the quantification of the TUNEL assay. 56% of the seminiferous tubules in the *Ctcf*-cKO mice contained on average three TUNEL-positive elongated spermatids, while no TUNEL-positive elongated spermatids were identified in wild-type or heterozygote testis. Two mice of each genotype were analyzed. Scale bars represent 100 micrometers.

We next analyzed if depleted CTCF levels in spermatocytes and spermatids in *Ctcf*-cKO mice had an effect on spermatogenesis. *Ctcf*-cKO mice were found to have five-fold smaller testis compared to their wild-type littermates and were infertile (Fig. 1A,B). Histochemical analysis of testis sections stained with hematoxylin and eosine (HE) showed that while formation of spermatocytes and round spermatids was unaffected in *Ctcf*-cKO testes, formation of elongated spermatids was defective (Fig. 1C). Analysis of the seminiferous tubules of *Ctcf*-cKO mice showed that 56% of them contained on average three TUNEL-positive elongated spermatids (Fig. 1D), suggesting that they were being eliminated through an apoptotic process. Mice heterozygous for *Ctcf* displayed reduced testis size but were fertile (Fig. 1A,B). Furthermore, histological analysis did not reveal obvious defects in testis morphology and apoptotic elongated cells were not observed in heterozygotes mice (Fig. 1C,D). Thus, spermiogenesis is severely affected in *Ctcf*-cKO mice.

### Elongated spermatids display aberrant head structures and irregular chromatin compaction in *Ctcf*-cKO testis

**Figure 2:**
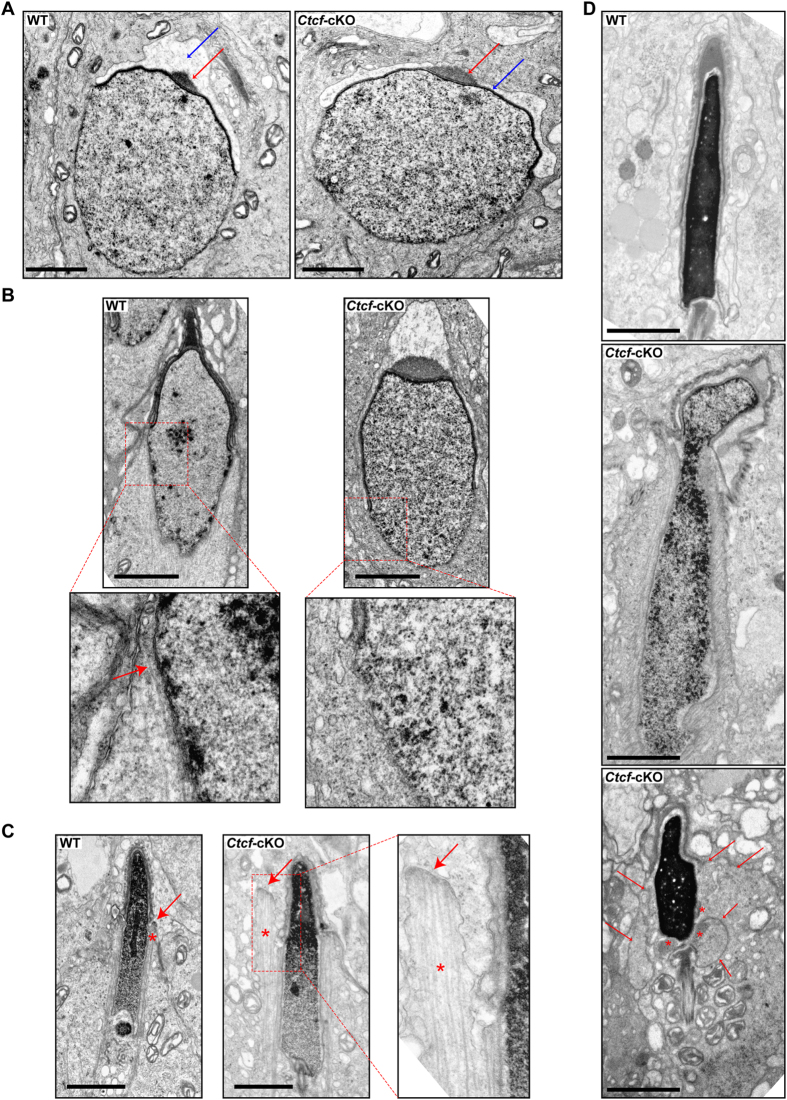
Elongated spermatids show chromatin compaction and sperm head defects in in *Ctcf*-cKO mice. (**A**) Round spermatids at stages 5–7 of spermiogenesis in wild-type (WT, left) and *Ctcf*-cKO mice (right). The arrows indicate the acrosome (red arrows) and the acrosome head cap (blue arrows). (**B**) Elongating spermatids at stages 8–10 of spermiogenesis in wild-type (WT, left) and *Ctcf*-cKO mice (right). Enlarged images of regions of spermatids at which manchette formation takes place are shown. Manchette formation appears to be impaired or delayed in *Ctcf*-cKO spermatids. The red arrow points to the microtubules of the manchette in the wild-type spermatid. (**C**) Elongating spermatids at stages 12–14 of spermiogenesis in wild-type (WT, left) and *Ctcf*-cKO mice (right). Microtubules (asterisk) of the normal manchette (arrow) in wild-type elongated spermatids are indicated. An abnormal manchette structure was apparent in *Ctcf*-cKO spermatids. A higher magnification of the manchette structure seen in the *Ctcf*-cKO spermatids, shows that the microtubules (asterisk) that constitute the manchette (arrow) display an aberrant organization relative to the sperm head. (**D**) Elongated spermatids at stages 15–16 of spermiogenesis in wild-type (WT, top) and *Ctcf*-cKO mice (second and third pictures from the top). *Ctcf*-cKO spermatids showed irregularities in chromatin compaction within the sperm head (middle picture), decompacted chromatin that protruded from the sperm head sperm (lower picture, asterisk) and head defects with a discontinuous nuclear membrane (lower picture, arrows). Scale bars represent 2 micrometers.

In order to understand how CTCF contributes to the differentiation process that transforms round haploid cells into spermatozoa, the ultrastructural organization of round, elongating and elongated spermatids in the testis of *Ctcf*-cKO mice was studied using transmission electron microscopy. We found the ultrastructural organization of round spermatids at steps 5–7 of spermiogenesis in wild-type and *Ctcf*-cKO mice to be very similar, including formation of an acrosome (Fig. 2A) and a chromatoid body in these cells (Supplementary Fig. S3). The first differences in the spermatid differentiation process in mutant and wild-type testis were observed at steps 8–10 of spermiogenesis. We found the manchette, a microtubule-containing structure^22^, to be distributed along the caudal, dorsal and ventral sides of the acrosome in elongating spermatids in *Ctcf*-cKO mice, whereas the manchette was localized to the caudal side of the acrosome in wild-type spermatids (Fig. 2B,C). Elongated spermatid heads in *Ctcf*-cKO mice at steps 12–14 of spermiogenesis, also frequently showed changes in nuclear morphology and chromatin compaction, relative to the organization of the same structures in wild-type elongated spermatids (Fig. 2D). In addition, weakly stained chromatin structures were frequently found to protrude out from a nuclear mass of strongly stained chromatin in mutant elongated spermatid heads (Fig. 2D). Thus, CTCF contributes to several critical aspects of spermiogenesis.

### PRM1 incorporation into chromatin is deficient in *Ctcf*-cKO mice

**Figure 3:**
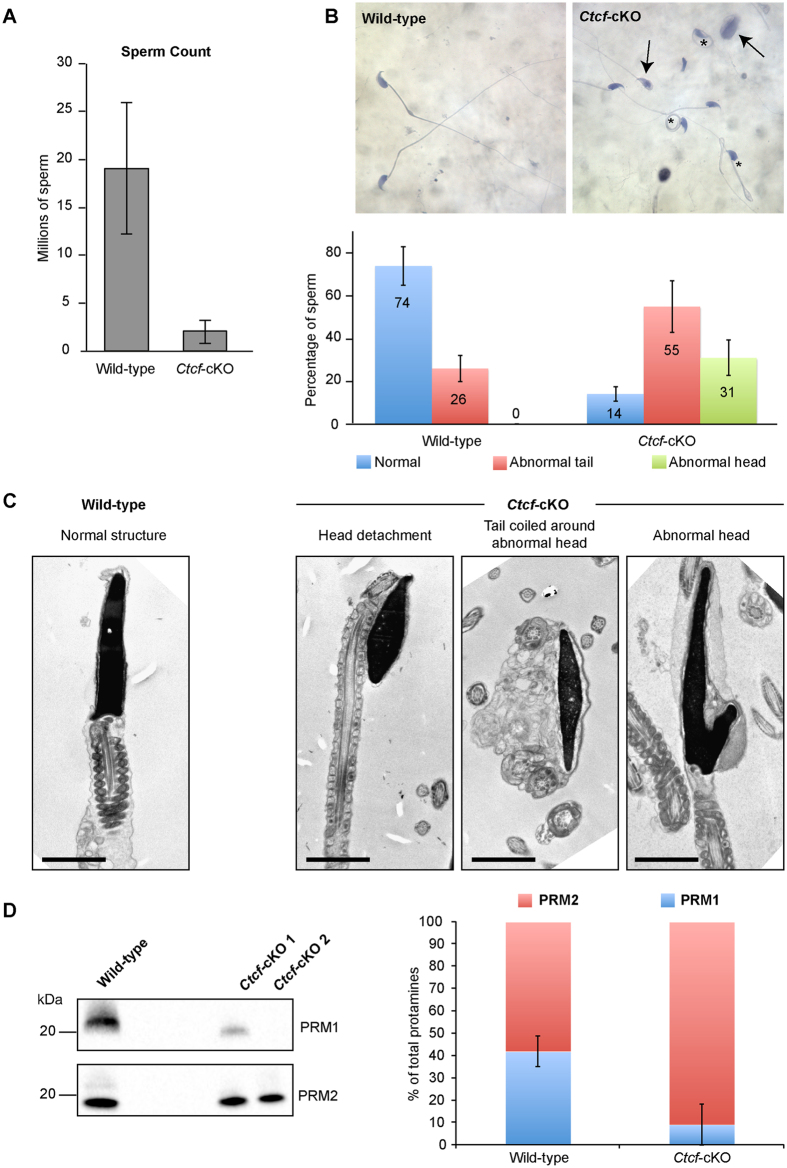
Mature spermatozoa in *Ctcf*-cKO mice display aberrant head and tail morphology and low protamine 1 levels. (**A**) The sperm count was reduced in *Ctcf*-cKO mice. Sperm from cauda epididymis for three wild-type and 12 *Ctcf*-cKO mice were analyzed. (**B**) Brightfield images of wild-type and *Ctcf*-cKO sperm. Aberrant head (arrows) and tail structures (asterisks) are found in spermatozoa in *Ctcf*-cKO mice. The results are summarized in a table below the brightfield images. Percentages of sperm from three biological replicates are indicated within the bars of the plot. (**C**) Electron microscopy analysis of *Ctcf*-cKO spermatozoa identified aberrant head and tail structures. Left, a wild-type sperm head showing normal head structure and tail attachment. Right, representative aberrant structures observed in the *Ctcf*-cKO spermatozoa. Scale bars represent 2 micrometers. (**D**) Reduction in protamine 1 (PRM1) levels in *Ctcf*-cKO sperm. Representative western blot of nuclear proteins from cauda epididymis sperm in one wild-type mice and two *Ctcf*-cKO mice. Right panel shows the quantification of western blot signals from three biological replicates. Immonoblotting of both protamines was done on the same membrane with an intermediate striping step, allowing comparing the relative percentages of the protamines in the same sample. The intensity of the two bands (PRM1 and PRM2) was set to 100% and used to obtain the relative percentage of the individual bands in the graph, showing and imbalance of the PRM1/PRM2 ration in *Ctcf*-cKO sperm. The PRM2 band was also used as indicator for loading control.

Mature spermatozoa are released into the cauda epididymis following completion of spermiogenesis^23^. We found that C*tcf*-cKO mice had a reduced amount of sperm in the cauda epididymis compared to wild-type mice (Fig. 3A). Histochemical and electron microscopy analysis of *Ctcf*-cKO sperm in cauda epididymis, revealed that one-third displayed abnormal head structures and more than 50% of the sperm had abnormal tail structures (Fig. 3B,C).

Mice deficient for PRM1 and PRM2 show deficiencies in sperm formation^7^,^8^,^24^, deficiencies similar to the ones observed in *Ctcf*-cKO mice. To find out if PRM1 and PRM2 levels were affected in *Ctcf*-cKO mice, nuclear protein extracts prepared from sperm isolated from cauda epididymis were analyzed by immunoblotting using antibodies against PRM1 and PRM2. We found the protein levels of PRM1 to be reduced in *Ctcf*-cKO sperm compared to wild-type sperm, whereas the protein levels of PRM2 seem unperturbed, producing an imbalance of the PRM1/PRM2 ratio (Fig. 3D). The levels of PRM1 and PRM2 in wild-type and *Ctcf*-cKO mice were also analyzed by immunolabeling of testis sections. The PRM1 antibody labeled all elongated spermatids in wild-type testis, whereas in the *Ctcf*-cKO testis elongated spermatids were weakly labeled or not labeled. Again, no differences in the staining pattern for PRM2 in the testis of *Ctcf*-cKO and wild-type mice were observed (Supplementary Fig. S4). Our results show that expression and/or incorporation of PRM1 in elongated spermatids in *Ctcf*-cKO mice is impaired.

### Expression of spermiogenesis-associated genes is down-regulated in *Ctcf*-cKO mice

It has been shown by ChIP-seq analysis that CTCF is bound to promoters of many genes in round spermatids in wild-type testis^18^. In order to evaluate if CTCF depletion abolishes expression levels of *Prm1*, *Prm2* or other spermiogenesis genes, we performed RNA expression microarrays. We found, using a 2-fold or greater expression change cutoff (p ≤ 0.05), 2549 coding genes to be down-regulated and 1557 coding genes to be up-regulated in the *Ctcf*-cKO testis (Supplementary Fig. S5, Supplementary Data S1 and S2). Comparison of the down-regulated and up-regulated coding genes in *Ctcf*-cKO testis with gene expression data from wild-type mouse staged cell populations (GSE21447 in the GEO database), showed that 84% of the down-regulated genes and 19% of the up-regulated genes were predominantly expressed in round spermatids (Supplementary Fig. S5). Interestingly, neither the *Prm1* nor the *Prm2* genes, both genes being expressed in round spermatids, were found to be down-regulated in *Ctcf*-cKO testis (Supplementary Data S3).

Functional analysis of the down-regulated coding genes in the *Ctcf*-cKO testis, using the functional annotation clustering analytical module from the DAVID bioinformatics resources^25^,^26^, identified a cluster of genes contributing to sexual reproduction, spermatogenesis and sperm formation (Supplementary Data S4). This cluster included genes known to affect the structural organization of elongated spermatids and spermatozoa, for example *H1fnt*, *Hook1*, *Spem1*, *Spata16*^27^,^28^,^29^,^30^,^31^ (Supplementary Data S5). Functional clustering of coding genes that were up-regulated in the *Ctcf*-cKO testis, showed the most significant annotation clusters to correspond to genes associated with cellular components, such as lysosomes, vacuoles and extracellular regions, and to biological processes related to responses to hormones and endogenous stimuli (Supplementary Data S6). The coding genes being up-regulated in the *Ctcf*-cKO testis were not further analyzed here.

In order to evaluate if down-regulation of gene expression in spermatids in *Ctcf*-cKO mice is linked to CTCF presence at the corresponding promoters (plus/minus 2 Kb around the TSS) or enhancers (2–20 Kb upstream of the TSS), we performed a comparison of the CTCF ChIP-seq data (GSE70764) to the data set GSE21447 in the GEO database and to our microarray expression assay. Our analysis identified 1289 and 2867 genes that had CTCF bound to the promoters or enhancers, respectively, and that were predominantly expressed in round spermatids (Supplementary Data S7). We found, however, only 169 genes (13% of the 1289 genes with CTCF on promoters) and 376 genes (13% of the 2867 genes with CTCF on enhancers) to be down-regulated in *Ctcf*-cKO testis (Supplementary Fig. S5 and Supplementary Data S7). Our results therefore show that in most cases the expression of genes with CTCF bound to their promoters in round spermatids, is not affected in *Ctcf*-cKO mice. Furthermore, a large majority of the genes found to be down-regulated in spermatids in *Ctcf*-cKO mice, do not have CTCF bound to their promoters or enhancers. Thus, down-regulation of gene expression in spermatids in *Ctcf*-cKO mice is in most cases not a result of CTCF binding to the promoters or enhancers of the genes being down-regulated. It is instead likely that the observed changes in gene expression in spermatids in *Ctcf*-cKO mice are caused by aberrant chromatin organization in mutant sperm, resulting from CTCF depletion.

### Histone retention in mature sperm is disrupted in *Ctcf*-cKO mice

**Figure 4:**
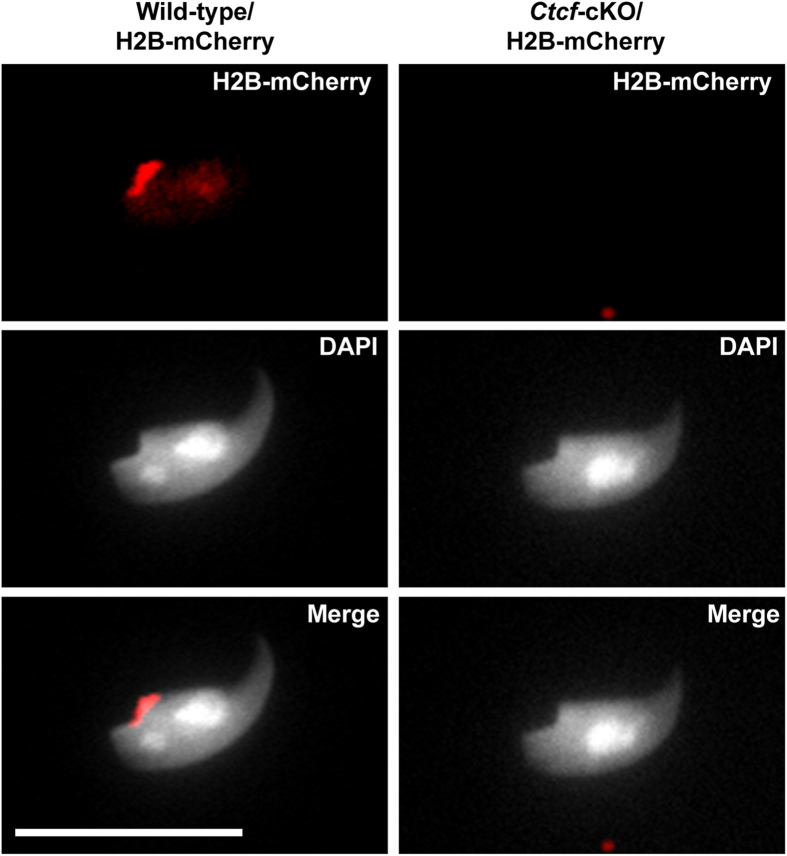
Histone retention defects in mature sperm of *Ctcf*-cKO testis. Histone H2B-mCherry localizes to the posterior region of the mature sperm head of wild-type/H2B-mCherry mice. Histone H2B-mCherry signal is absent from the mature sperm head of *Ctcf*-cKO/H2B-mCherry mice in 56% of the analyzed sperm. Calibration bar represents 10 micrometers.

It has been shown that positions along chromosomes at which histones are retained in spermatozoa, frequently overlap with DNA binding motifs bound by CTCF^9^. We hypothesized that disrupted expression of CTCF during spermiogenesis, would impair histone retention in mature sperm. To monitor histone levels in mature sperm depleted for CTCF, the *Ctcf*-cKO strain was crossed with a mouse strain expressing a nuclear encoded histone H2B-mCherry fusion protein^32^, and mature sperm then were isolated from cauda epididymis. We found the histone H2B-mCherry fusion protein to be preferentially localized at the posterior region of the sperm head in wild-type mice (Fig. 4). In contrast, a majority (56%) of the sperm isolated from *Ctcf*-cKO/H2B-mCherry mice did not display a histone H2B-mCherry signal (Fig. 4), strongly indicating that CTCF contributes to histone retention in mature sperm.

### Meiotic progression is not affected in *Ctcf*-cKO mice

**Figure 5:**
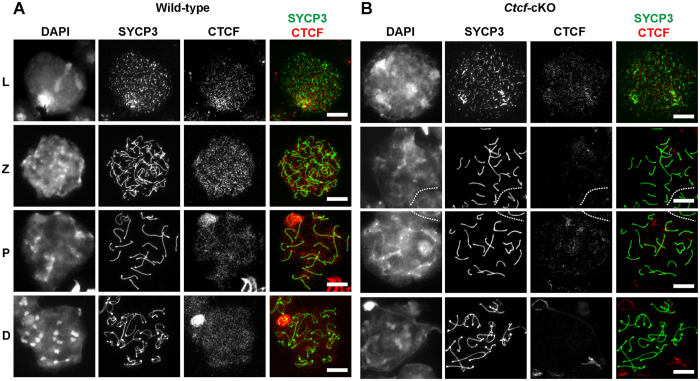
Localization of CTCF to the XY body is impaired in *Ctcf*-cKO meiotic cells. (**A**) Staining of spermatocytes at different stages of meiosis in wild-type testis using DAPI (stains DNA), a SYCP3 antibody and a CTCF antibody. SYCP3 labels the axis of meiotic chromosomes in a pattern unique to the different stages of prophase I, identifying spermatocytes at the leptotene (L), zygotene (Z), pachytene (P) and diplotene (D) stages of meiosis. The CTCF antibody preferentially labels the XY body in pachytene and diplotene meiotic cells in wild-type testis. (**B**) Staining of spermatocytes at different stages of meiosis in *Ctcf*-cKO testis using DAPI (stains DNA), a SYCP3 antibody and a CTCF antibody. A strong reduction of CTCF levels was observed in meiotic cells in *Ctcf*-cKO testes. Scale bars represent 10 micrometers.

**Figure 6:**
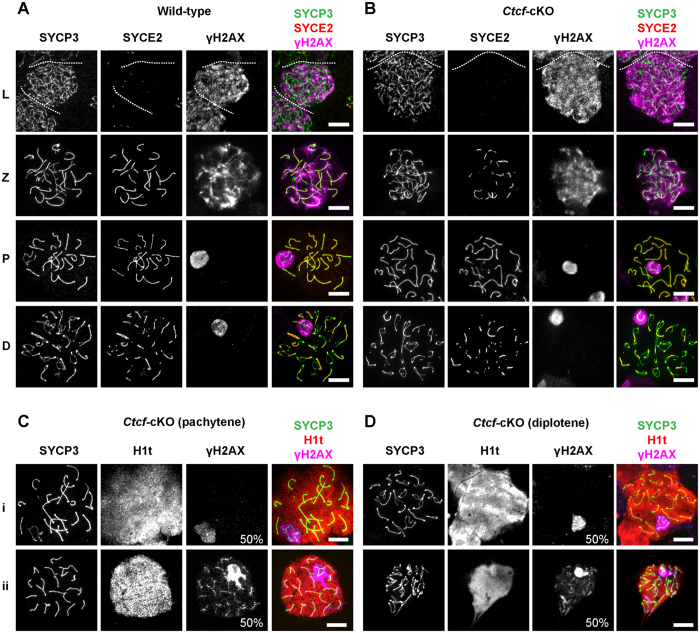
γH2AX staining patterns in spermatocytes from wild-type and *Ctcf*-cKO testis. (**A,B**) Labeling of wild-type and *Ctcf*-cKO spermatocytes with antibodies against SYCP3, SYCE2 and γH2AX. γH2AX labels DNA double strand breaks and the XY body, whereas SYCP3 and SYCE2 label the axis of homologous chromosomes and regions of synapsis between homologous chromosomes, respectively. Leptotene (L), Zigotene (Z), Pachytene (P) and Diplotene (D). (**C,D**) (i) Normal distribution of γH2AX at the XY body. (ii) Abnormal distribution of γH2AX at the XY body and automosomic axes in pachytene (around 50%) and diplotene (around 50%) spermatocytes of *Ctcf*-cKO testis. Labeling of spermatocytes at the pachytene and diplotene stages of meiosis was done using antibodies against SYCP3, histone H1t and γH2AX. H1t accumulates in spermatocytes at the midpachytene to diplotene stages of meiosis. Four mice of each genotype were used. Scale bars represent 10 micrometers.

The histochemical analysis of *Ctcf*-cKO testis revealed no abnormalities in the formation or the differentiation of spermatocytes (Fig. 1C). This was surprising, as immunostaining of testis sections from *Ctcf*-cKO mice showed CTCF expression to be abrogated in mutant spermatocytes (Supplementary Fig. S2). To further analyze this, spermatocytes were isolated from the testis of *Ctcf*-cKO and wild-type mice and immuno-labeled with an antibody against CTCF. We found that whereas CTCF labeled the XY body in spermatocytes in wild-type mice, no CTCF labeling of the XY body was observed in *Ctcf*-cKO spermatocytes (Fig. 5A,B). In order to find out if CTCF depletion had a more subtle effect on meiotic progression in spermatocytes, we used antibodies against the synaptonemal complex proteins SYCP3 and SYCE2, the cohesin complex proteins REC8, RAD21 and RAD21L and the recombination protein MLH1, as markers to monitor progression of meiosis in spermatocytes in *Ctcf*-cKO mice^33^,^34^,^35^,^36^,^37^,^38^,^39^,^40^. We observed no obvious differences in the expression patterns of these markers in *Ctcf*-cKO and wild-type spermatocytes, suggesting that neither the organization of the axes of meiotic chromosomes, formation of the synaptonemal complex, or the formation of mature recombination intermediates, were affected by depletion of CTCF (Fig. 6A,B, Supplementary Figs S6 and S7). The XY body in spermatocytes becomes transcriptionally silenced during meiosis^41^,^42^,^43^. The preferential localization of CTCF at the XY body in wild-type meiotic cells (Fig. 5A), suggested that CTCF could contribute to XY body formation. We found that 50% of the pachytene and diplotene spermatocytes in *Ctcf*-cKO mice displayed an abnormal γH2AX distribution pattern (Fig. 6C,D). However, analysis of the distribution patterns of proteins that contribute to the functional organization of the sex body, including ATR^44^, RNA Polymerase II^42^,^45^ and MDC1^46^, failed to identify any changes in the expression pattern of these proteins in *Ctcf*-cKO spermatocytes relative to wild-type spermatocytes (Supplementary Fig. S7). Furthermore, X-Y chromosome pairing was not affected in spermatocytes at the pachytene stage of meiosis in *Ctcf*-cKO mice (Supplementary Fig. S7). We found that the expression levels of coding genes preferentially expressed in primary spermatocytes in wild-type mice (e.g., *Stra8*, *Rad51*, *Mlh1*, *Rec8*, *Rad21*, *Rad21l*, *Sycp3*, *Syce2*, *Syce3*, *Tex12*), were not affected in *Ctcf*-cKO testis (Supplementary Data S3), in agreement with the lack of phenotypic changes observed in spermatocytes in *Ctcf*-cKO mice. In summary, our experiments do not reveal a significant role for CTCF during meiosis in *Ctcf*-cKO mice.

## Discussion

We have here analyzed the role of CTCF during male germ cell development. Conditional inactivation of the *Ctcf* gene in pre-leptotene spermatocytes drastically depleted CTCF protein levels in spermatocytes and spermatids and resulted in impaired spermiogenesis and infertility. Elongated spermatids in *Ctcf*-cKO mice showed aberrant chromatin compaction and manchette formation, whereas mature sperm displayed abnormal head and tail structures, and loss of histone retention. Thus, CTCF has an important role in the formation of mature germ cells.

Gene expression analysis, using RNA microarrays, identified a large number of genes that were down-regulated in the testis in *Ctcf*-cKO mice. A majority of the down-regulated genes were expressed in round spermatids, many of them contributing to the structural organization of spermatozoa in wild-type mice. We found that a large majority of the genes that were down-regulated in spermatids in *Ctcf*-cKO mice, did not have CTCF bound to their promoters or enhancers in spermatids in wild-type mice, and of the genes that had CTCF bound to their promoters or enhancers in spermatids in wild-type mice, only 13% (for both cases) were down-regulated in *Ctcf*-cKO mice. We therefore conclude that in a majority of cases, the observed changes in gene expression *Ctcf*-cKO mice, is likely to be caused by aberrant chromatin organization in mutant spermatids.

DNA compaction during spermiogenesis is dependent on the replacement of histones with sperm-specific protamines^4^,^24^. Haploinsufficiency for the *Prm1* gene in mice affects sperm head and tail morphology, as well as chromatin compaction, in spermatozoa isolated from the cauda epididymis, resulting in infertility^7^,^8^. We found the levels of PRM1 in spermatozoa to be sharply reduced in spermatozoa from *Ctcf*-cKO mice, whereas the levels of PRM2 appeared to be unaffected, resulting in a changed PRM1:PRM2 ratio. *Prm1* transcription levels were unaffected in the *Ctcf*-cKO mice, thus suggesting a post-transcriptional regulation of protamine deposition, as suggested for the weak immunolabaleling pattern of PRM1 on *Ctcf*-cKO testis sections. The similarities in the phenotypes seen for spermatozoa in *Ctcf*-cKO mice and PRM1 haploinsufficient mice, suggest that a reduced expression of PRM1 in *Ctcf*-cKO spermatozoa affects chromatin compaction, and as a consequence also manchette organization, sperm head and tail formation.

The sperm count in the cauda epididymis of *Ctcf*-cKO mice was reduced by approximately 90% relative to the situation in wild-type mice, whereas the sperm count in haploinsufficient PRM1 mice was reduced by approximately 35%^7^. The further reduced sperm count in *Ctcf*-cKO mice, compared to haploinsufficient PRM1 mice, could be a result of the lower PRM1 levels observed in *Ctcf*-cKO mice, relative to the situation in haploinsufficient PRM1 mice. Alternatively, the drastically reduced sperm count in *Ctcf*-cKO mice could result from the down-regulation of genes that take part in the structural organization of elongated spermatids and spermatozoa, for example *H1fnt*, *Hook1*, *Spem1*, *Spata16*^27^,^28^,^29^,^30^,^31^. Thus the observed reduced expression of these genes in spermatids is likely to add to the aberrant organization of spermatozoa in *Ctcf*-cKO mice.

A direct role of CTCF in histone retention on specific DNA sequences in mature mouse sperm has been suggested by the presence of nucleosomes at CTCF binding motifs in mature sperm^9^ and by the presence of CTCF on promoters in round spermatids, many of which show histone retention in mature sperm^18^. Core histones have been immunolocalized to the periphery of the mature mouse sperm nucleus^47^ while histone H4 and the testis specific histone H2B (TH2B) have been immunolocalized to the center of the sperm nucleus, overlapping with the DAPI-rich chromocenter^9^. Furthermore, the presence of all the five canonical histones in mature sperm has been detected by mass spectrometry^48^. We analyzed if CTCF depletion would impair histone retention in mature sperm, using a mouse strain expressing a nuclear encoded histone H2B-mCherry fusion protein^32^, this approach allowed us to monitor histone retention in mature sperm without the need of permeabilization and altering sperm structure to allow histone detection using antibodies. We found that while the histone H2B-mCherry fusion protein preferentially localized to the posterior region of the sperm head in wild-type mice, a majority (56%) of the sperm isolated from *Ctcf*-cKO/H2B-mCherry mice did not display a histone H2B-mCherry signal. Thus, CTCF depletion results in an accelerated loss of histone from chromatin in nuclei of mature sperm. CTCF is the only transcription factor that has been shown to produce well-positioned nucleosomes around its DNA binding sites^49^. This property could be important to retain specific histone variants during nucleosome replacement in elongating spermatids. Furthermore, a recent model proposes that resistance to load transition proteins prior to protamine deposition may be mediated by a DNA-binding protein that recognize unmethylated DNA sequences^10^, thus the ability of CTCF to bind preferentially to its unmethylated DNA-binding motif 50,51 makes CTCF a strong candidate to contribute to histone retention in elongating spermatids. Histone retention in mature sperm has been have been suggested to be a mechanism to transfer epigenetic memory from the sperm chromatin to the embryo^9^,^10^,^11^,^52^,^53^, thus our mouse model provides an opportunity to assay the effects of histone retention in mature sperm.

CTCF ha been shown to act as a global regulator of chromatin organization in somatic cells^15^,^17^. Brother of Regulator of Imprinting Sites (BORIS) arose from a gene duplication of *Ctcf* during early evolution in amniotes and its physiological expression is restricted to male germ cells and aberrantly expressed in some cancer cells^54^,^55^. Both proteins are expressed throughout spermatogenesis of mammals, although the detailed expression pattern of BORIS is still debated^18^,^21^,^55^. Analysis of *BORIS*-KO mice has discovered BORIS to be dispensable for mice fertility, revealing only a small reduction in the number of round spermatids^56^. We show here that *Ctcf*-cKO mice display infertility, a drastic reduction of testis weight, low mature sperm counts, severe structural defects in elongated spermatids and mature sperm, and down-regulation of genes in spermatids required for formation of sperm. Therefore CTCF, but not BORIS, contribute in a critical way to sperm fertility in male mice.

## Methods

### Generation of a male germ line-specific conditional knockout mouse model

Animal care and methods described here were carried out in accordance with the regulations set up by the Swedish National Board of Agriculture. Experimental protocols were approved by the Karolinska Institutet biosafety committee and the Swedish National Board of Agriculture. The mice used in the study had a C56BL/6J background. A mouse strain with floxed *Ctcf* alleles^19^ (*Ctcf*^*f/f*^) was crossed with a Stra8-cre (*Stra8-iCre*) transgenic mouse strain (Tg(Stra8-cre)1Reb/J from the Jackson laboratory). The *Stra8-iCre* transgene effectively targets genes at the pre-leptotene stage of meiosis I^20^, and reach full penetrance at the pachytene stage^57^ in male mice. To maximize the efficiency of the *Stra8-iCre* transgene^58^, we used a heterozygous mouse strain in which one copy of *Ctcf* gene was excised, leaving one copy being floxed (*Stra8-iCre-Ctcf*^*f/Δ*^ or *Ctcf*-cKO). We used mice that were 12 to 15 weeks old to minimize age-related variations. We compared the *Ctcf*-cKO (*Stra8-iCre-Ctcf*^* f*/*Δ*^) strain to the wild-type littermates of genotype *ctcf*^* f /f*^, *ctcf*^* wt/f*^ or *ctcf*^* wt/wt*^ and to the heterozygous littermates of genotypes *Stra8-iCre-ctcf*^* f/wt*^, *Stra8-iCre-ctcf*^*Δ*/wt^, *ctcf*^* f*/*Δ*^ or *ctcf*^*Δ*/*wt*^. No difference in body or testes size was observed for wild-type (*Ctcf *^*wt/wt*^) relative to strains having one wild-type and one floxed *Ctcf* allele (*Ctcf*^* wt/f*^) or strains having both *Ctcf* alleles floxed (*Ctcf*^* f/f*^) and these mouse strains were therefore referred to as “wild-type”. To generate the *Ctcf*-cKO mice strain in a H2B-mCherry genetic background, we crossed heterozygous mice of the *Ctcf*-cKO strain with homozygous mice of the reporter mice strain R26-H2B-mCherry^32^ (CDB accession number: CDB0239K, http://www.cdb.riken.jp/arg/mutant%20mice%20list.html) for several generations until obtain *Ctcf*-cKO/H2B-mCherry mice.

### Histologic analysis and Immunofluorescence

Testes were prepared for immunohistochemistry by fixing with Histochoice (Electron Microscopy Science), dehydrated and paraffin embedded. Sections (6 μm thick) were mounted on glass slides stained with hematoxylin and eosine or processed for immunostaining. For Immunostaining, antigen retrieval was performed using an antigen retrieval citra plus method (BioGenex), according to the manufacturer’s instructions. Samples were then subjected to immunostaining. Nuclear spreads of testicular cells were performed as previously described^59^. The following antibodies and dilutions were used: mouse anti-SYCP3 (Santa Cruz Biotechnology), 1:400; rabbit anti-CTCF (Upstate), 1:400; guinea pig anti-SYCE2^60^, 1:200; rabbit anti-γH2AX (Upstate Biotechnology), 1:100; guinea pig anti-histone H1t (kindly provided by N. Hunter), 1:1000; monoclonal mouse anti-Hup 1N (Protamine 1, Briar Patch Biosciences LLC), 1:200; monoclonal mouse anti-Hup 2B (Protamine 2, Briar Patch Biosciences LLC), 1:200; goat anti-ATR (Santa Cruz Biotechnology), 1:100; rabbit anti-MDC1 (Abcam), 1:100; rabbit anti-RAD21 (Abcam), 1:200; rabbit anti-REC8^38^, 1:200; rabbit anti-RAD21L^38^, 1:200; guinea pig anti-REC8^60^, 1:200; guinea pig anti-SYCE1^60^, 1:500 and mouse anti-MLH1 (Pharmigen), 1:50. Signals were visualized using secondary antibodies as follows: donkey anti-mouse Alexa Fluor 488 (Invitrogen), 1:1000; donkey anti-mouse Cy2 (Jackson ImmunoResearch), 1:200; donkey anti-guinea pig Cy3 (Jackson ImmunoResearch), 1:600–1:1000; swine anti-rabbit FITC (DakoCytomation), 1:400; and donkey anti-rabbit Cy5 (Jackson ImmunoResearch), 1:1000. Stained slides were mounted and DAPI-stained using ProLong Gold (Invitrogen). Image acquisition of a single focal plane was done using a Leica microscope with a Hamamatsu camera and Openlab 3.1.4 software (Improvision). Image processing and analysis was carried out using the Volocity soft ware package (Perkin Elmer). FISH was performed according to the protocol provided by the probe manufacturer using a chromosome paint probe for chromosome Y together with a point-probe hybridizing with DXMit190 loci located on chromosome X (ID Labs Inc.). Spermatocytes were staged according to DNA morphology and SYCP3 staining.

### TUNEL assay

Testis sections were labeled according to the manufacturer’s instructions (ApopTag Red Insitu Apoptosis kit, Milliport). Seminiferous tubules, sectioned transversally, with TUNEL positive and TUNEL negative elongated cells were counted. In total 79, 142 and 261 transversally sectioned seminiferous tubules from two wild-type, two heterozygote and two *Ctcf*-cKO mice were used for analysis, respectively. The number of TUNEL positive elongated spermatids within every tubule was used to obtain the average of TUNEL positive elongated spermatids per positive-labeled tubule.

### Electron microscopy

Small pieces of testis were fixed in 2.5% glutaraldehyde plus 4% PFA in cacodylate buffer (pH7.2). After postfixation with 2% OsO4, pre-embedding staining was performed with 0.5% uranyl acetate. Samples were dehydrated through graded ethanol solutions, embedded in epoxic-resin durcupan ACM (Electron Microscopy Sciences) and polymerized for 48 hrs at 60 °C. Sample preparation of epididymal sperm was performed as previously reported^61^. 80 nm ultra-thin sections were collected on formvar/carbon-coated one-slot copper grids (Agar scientific), contrasted with uranyl acetate and lead citrate before examination in a transmission electron microscope (Philips, CM120) with a voltage acceleration of 100 kilovolts.

### Immunoblotting

Testes were homogenized in a buffer containing 0.32 M sucrose, 10 mM HEPES (pH 7.4), 1 mM phenylmethylsulfonyl fluoride (PMSF), and the complete protease inhibitor cocktail (Roche). After centrifugation at 1000 × *g*, the supernatant was collected as cytoplasmic extracts. The pellet was resuspended in RIPA buffer (50 mM Tris–HCl (pH 7.5), 150 mM NaCl, 1 mM EDTA, 1% NP-40, 0.5% Na-deoxycholate, 0.1% SDS, and protease inhibitors). After sonication and centrifugation at 16000 × *g*, the supernatant was recovered as nuclear extracts. Extracts were separated on a 4–15% Mini-PROTEAN TGX Stain-Free Precast Gels (BioRad) in Tris–glycine running buffer and were subsequently transferred onto PVDF membranes with the Trans-Blot Turbo Transfer System (BioRad). To detect CTCF and Lamin B1 the following antibodies and dilutions were used: rabbit anti-CTCF (Upstate), 1:1000; and goat anti-Lamin B1 (M20) (Santa Cruz Biotechnology), 1:300. Signals were detected with horseradish peroxidase-conjugated secondary antibodies and visualized by ECL Prime (GE Healthcare). The quantitative evaluation of the bands was carried out with Image Lab software (BioRad).

### Analysis of sperm from the cauda epididymis

The cauda epididymides from wild-type, *Ctcf*-cKO and *Ctcf*-cKO/H2B-mCherry mice were placed on a petri dish with 300 μl of PBS and cut into smaller pieces to release sperm. After 30 min of incubation, the sperm containing solution was collected. Small aliquots of the sperm solution (5 μl) were taken for sperm counts and the rest of the solution was diluted 1:1 with a 4% PFA solution. 100 μl of sperm solution was placed on poly-l-lysine covered slides and air-dried for 30 min. Dried slides were mounted with ProLong Gold antifade reagent with DAPI (Invitrogen) or stained with hematoxylin-eosine and visualized under florescence microscopy or bright filed microscopy.

### Extraction of sperm nuclear proteins from cauda epididymis sperm and immunoblotting

The cauda epididymis from wild-type or *Ctcf*-cKO mice were placed on a petri dish with 60 μl of Hanks’ balanced salt solution (HBSS, SIGMA) and cut into smaller pieces to release sperm. After 30 min of incubation, the sperm-containing suspension was collected and sperm counting was done. Protein extraction was performed on fresh or frozen samples of sperm suspensions. Sperm tail dissociation and sperm nuclear protein extraction were performed as previously described^62^. For immunoblotting, the protein pellets were resuspended in Laemmli sample buffer and boiled at 90 °C for 10 min. Protein separation, transfer and immunoblotting were done as described above. To detect protamines 1 and 2, the following antibodies and dilutions were used: monoclonal mouse anti-Hup 1N (Briar Patch Biosciences LLC), 1:1000; and monoclonal mouse anti-Hup 2B (Briar Patch Biosciences LLC), 1:1000.

### Microarray and gene ontology analysis

Seminiferous tubules of wild-type and *Ctcf*-cKO mice were placed in RNA stabilization reagent RNAlater (QIAGEN). RNA was extracted and purified with the RNeasy kit (QIAGEN). Three independent wild-type and four independent *Ctcf*-cKO microarray analysis were performed using the GeneChip mouse transcriptome assay 1.0 (Affimetrix). Analysis of the microarray data was done according to Affimetrix guidelines. The microarray data passed all the quality controls of the Expression Console (Affimetrix) and transcriptional changes were monitor using the Transcription Analysis Console (Affimetrix). Microarray data have been deposited in the Gene Expression Omnibus database (GEO) with the following accession number: GSE76439. Analysis of the wild-type expression patterns of the miss-regulated coding genes in the *Ctcf*-cKO testis was done as previously reported^63^ using microarray gene expression data from FACS sorted testis populations that has been deposited in the Gene Expression Omnibus database (GSE21447)^64^ and visualized with the Qlucore Omics Explorer 3.1 (Qlucore AB, Sweden). Database for annotation, visualization and integrated discovery (DAVID) gene ontology (GO) analysis was performed using the DAVID Bioinformatics Resources database^25^,^26^.

### CTCF occupancy on gene promoters or enhancers

Genomic coordinates for the promoters (defined as plus/minus 2 Kb around the TSS^17^,^18^) and enhancers (defined as 2–20 Kb upstream of the TSS^17^) from the down-regulated, up-regulated (in the *Ctcf*-cKO testis) and genome-wide coding genes were based on the mouse mm9 assembly (July 2007 Build 37 assembly by NCBI and Mouse Genome Sequencing Consortium) and downloaded from the UCSC table browser (http://genome.ucsc.edu/)^65^. For each gene, coordinates corresponding to the canonical isoform were selected with the KnownCanonical table from the associated tables of the Table Browser. Files with the gene and promoter genomic coordinates were intersected with the genomic coordinates of CTCF occupancy in round spermatids (CTCF-Narrow-peaks-ChIP-seq data) from the GEO database (GSE70764)^18^ using BEDtools suite^66^. Intersection files were then visualized and analyzed with the Galaxy web-based platform (usegalaxy.org)^67^,^68^,^69^.

### Other methods

FACS sorting of testicular cells was done according to standard protocols^70^.

## Additional Information

**How to cite this article**: Hernández-Hernández, A. *et al*. CTCF contributes in a critical way to spermatogenesis and male fertility. *Sci. Rep.* **6**, 28355; doi: 10.1038/srep28355 (2016).

## Supplementary Material

Supplementary Information

Supplementary Dataset 1

Supplementary Dataset 2

Supplementary Dataset 3

Supplementary Dataset 4

Supplementary Dataset 5

Supplementary Dataset 6

Supplementary Dataset 7
