# Supplementary material for: CTCF contributes in a critical way to spermatogenesis and male fertility
Source: Sci Rep. 2016 Jun 27;6:28355. doi: 10.1038/srep28355 (PMC4921845; doi:10.1038/srep28355)
Supplement: Supplementary Information [file srep28355-s1.pdf]

CTCF contributes in a critical way to spermatogenesis and male fertility

Abrahan Hernández-Hernández[1]*, Ingrid Lilienthal[1], Nanaho Fukuda[1,2], Niels Galjart[3] and Christer Höög[1]

[1]Karolinska Institutet, Department of Cell and Molecular Biology, Berzelius väg 35, 171 77 Stockholm, Sweden

[2]Current address: Graduate School of Biological Sciences, Nara Institute of Science and Technology, 8916-5 Takayama, Ikoma, Nara 630-0192 Japan

[3]Department of Cell Biology and Genetics, Erasmus MC, 2040 CA Rotterdam, The Netherlands

*Corresponding author: e-mail address: abrahan.hernandez@ki.se

Department of Cell and Molecular Biology,

Karolinska Institutet,

Berzelius väg 35, 171 77 Sweden.

Tel. +46 8 52487396

Supplementary Figures S1 – S7

Supplementary Data S1- S7

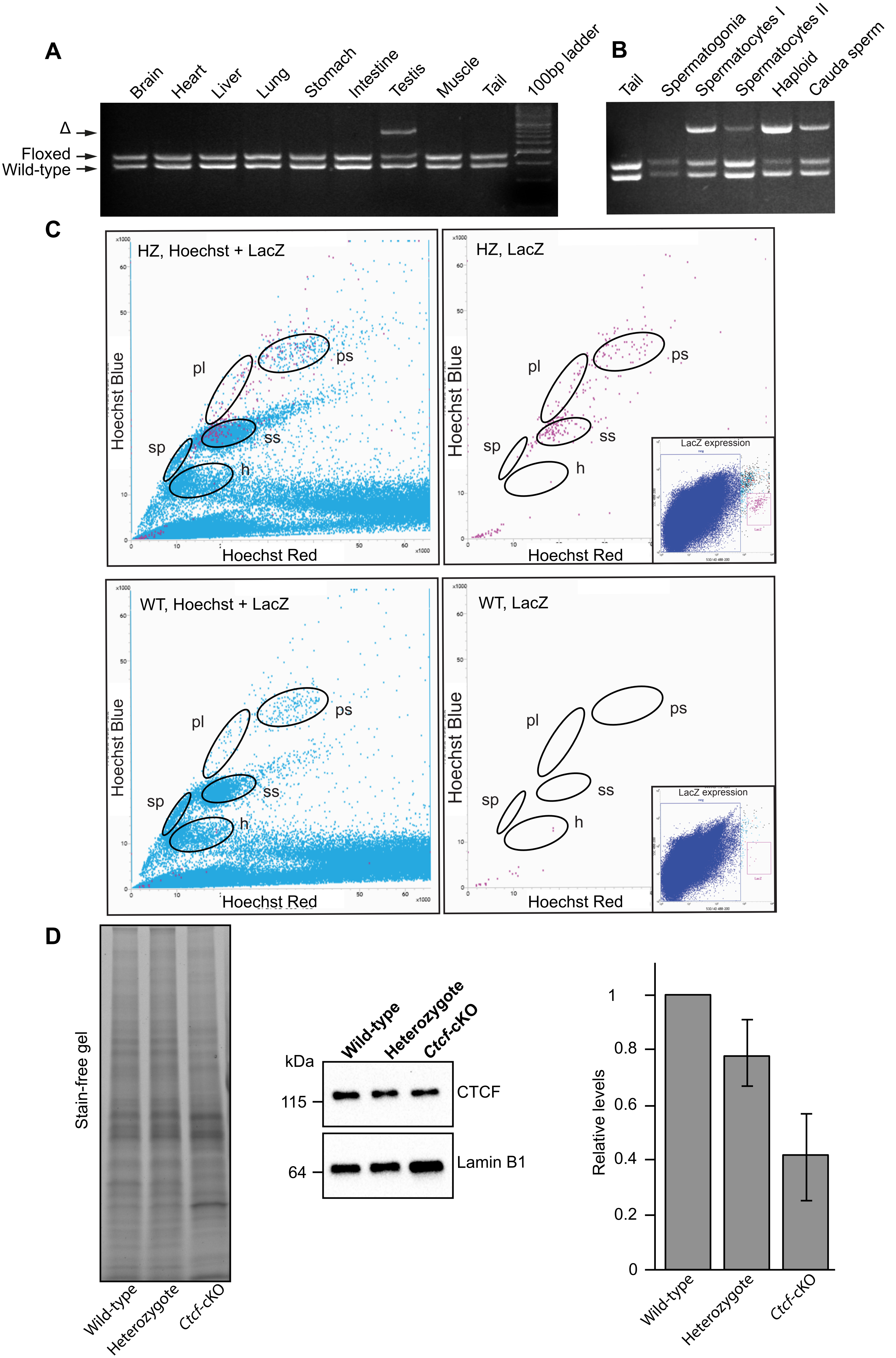
A
Brain
Heart
Liver
Lung
Stomach
Intestine
Testis
Muscle
Tail
100bp ladder
Δ
Floxed
Wild-type
B
Tail
Spermatogonia
Spermatocytes I
Spermatocytes II
Haploid
Cauda sperm
C
HZ, Hoechst + LacZ
HZ, LacZ
WT, Hoechst + LacZ
WT, LacZ
Hoechst Blue
Hoechst Red
pl
ps
sp
ss
h
LacZ expression
D
Stain-free gel
Wild-type
Heterozygote
Ctcf-cKO
kDa
115
64
CTCF
Lamin B1
Relative levels
1
0.8
0.6
0.4
0.2
0

Supplementary Figure S1. Conditional inactivation of the *Ctcf* gene in spermatocytes

(**A**) PCR analysis of different tissue types from mice heterozygous for the *Stra8-iCre* conditional *Ctcf* allele (*Stra8-iCre-Ctcf* $^{wt/f}$) revealed that PCR bands representing the deleted gene (Δ) appeared only in testis. The bands representing floxed and wild-type alleles are indicated. (**B**) PCR analysis of FACS-sorted cells from testis of the *Stra8-iCre-Ctcf* $^{wt/f}$ strain. (**C**) FACS profiles showing Hoechst staining and LacZ expression. Merged Hoechst and LacZ profiles (left panel) and the LacZ profile alone (right panel) of testes from *Stra8-iCre-Ctcf* $^{wt/f}$ mice (HZ) and wild-type (W) are shown. Cell types indicated on the FACS profiles are: sp, spermatogonia; h, haploid; ss, secondary spermatocytes; pl, pre-leptotene; ps, primary spermatocytes. (**D**) Immunoblot analysis of CTCF nuclear levels in testis from wild-type, heterozygous and *Ctcf*-cKO mice (middle panel). Five micrograms of protein for every mouse genotype were loaded in each gel well. Stain-free polyacrylamide gel was used to visualize similar protein loading conditions (left panel). Anti-lamin B, that is expressed throughout spermatogenesis in rodents[71] was used as additional loading control. The right panel displays the relative quantification of the CTCF/Lamin B1 band intensities in the immunoblots from seven biological replicates.

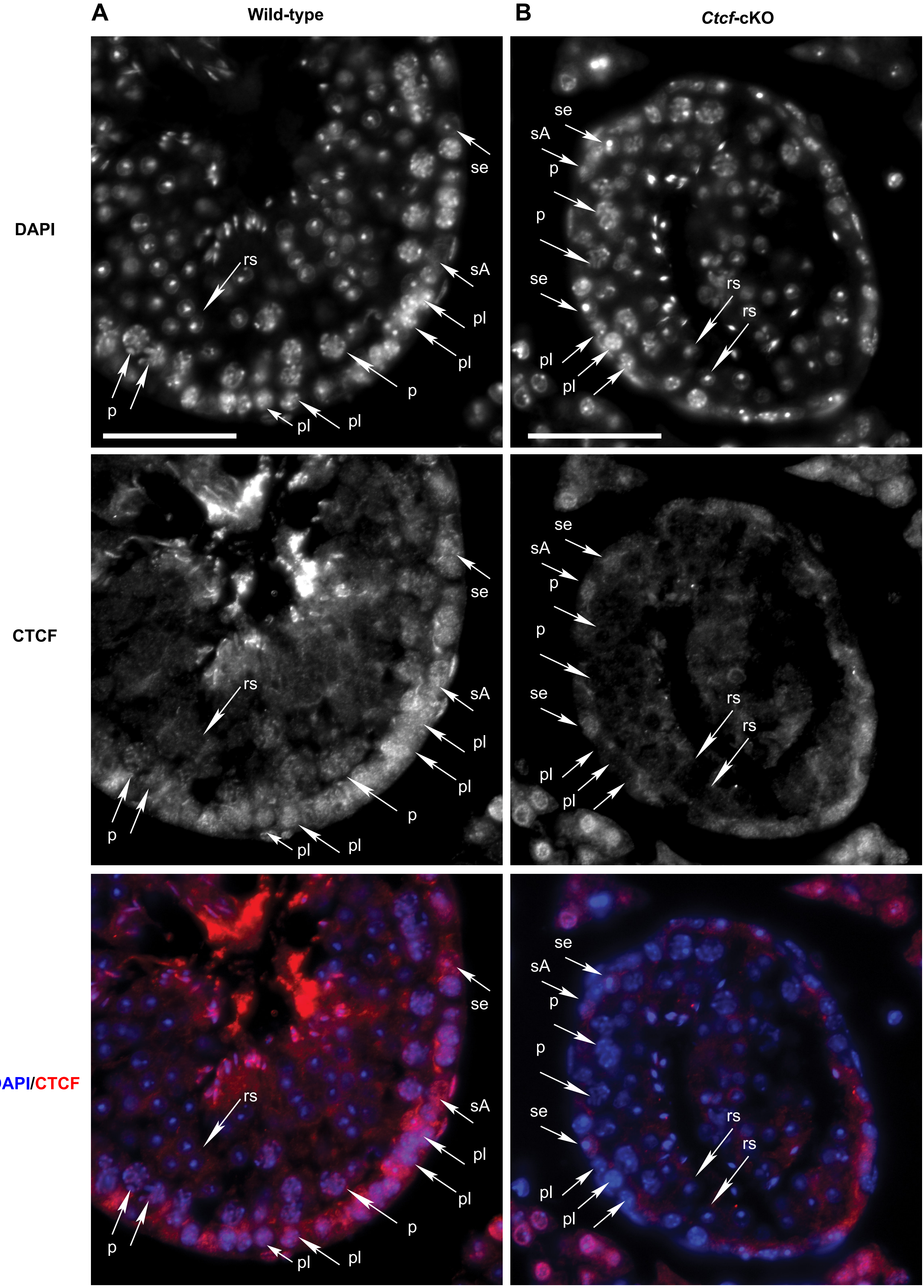
A
Wild-type
B
Ctcf-cKO
DAPI
CTCF
DAPI/CTCF
se
sA
pl
p
rs

Supplementary Figure S2. CTCF expression is reduced in *Ctcf*-cKO testis

(**A-B**) Immuno-histological analysis of the abundance of CTCF in sections of paraffin-embedded testes of wild-type and *Ctcf*-cKO mice. Cell types in the sections are indicated as follows: p, pachytene; pl, pre-leptotene; sA, spermatogonia type A; se, Sertoli cell; rs, round spermatid. Scale bar represent 50 micrometers.

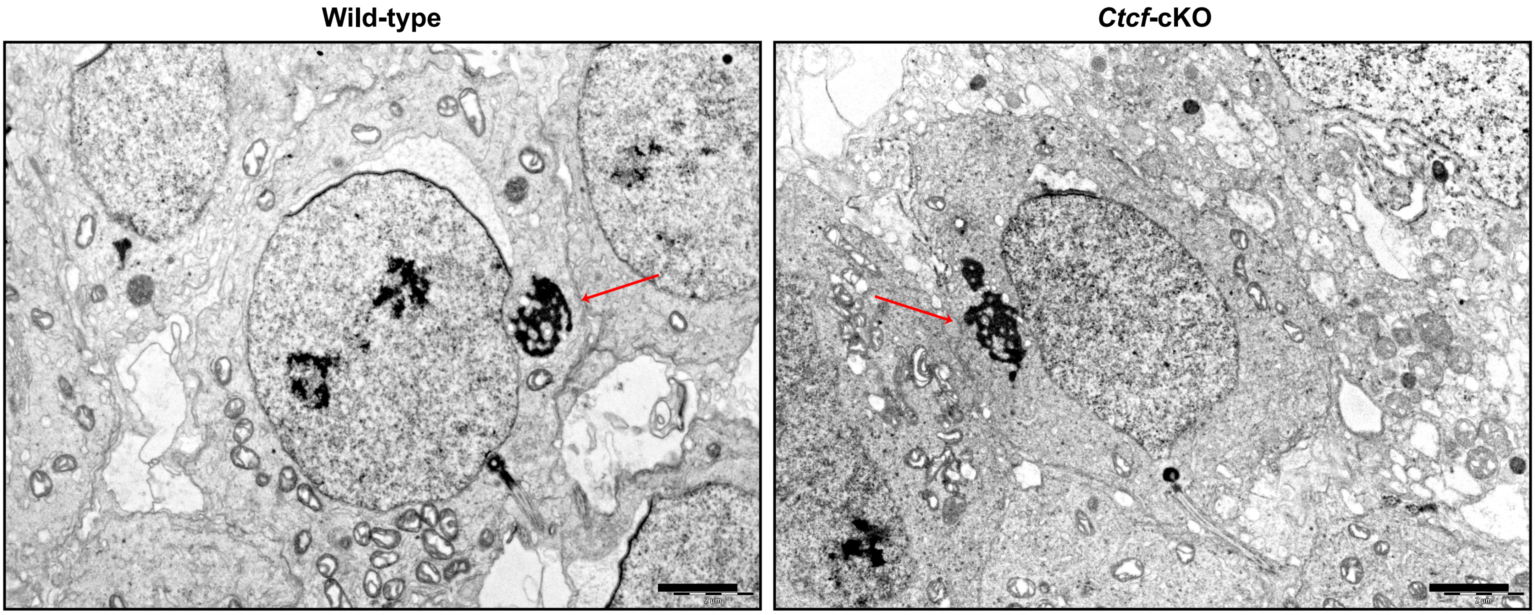
Wild-type
Ctcf-cKO

Supplementary Figure S3. The chromatoid body is properly formed in round spermatids cells in *Ctcf*-cKO mice.

Electron microscopy analysis of round spermatids at stages 5-7 identified a chromatoid body (a perinuclear granule) in wild-type, as well as in *Ctcf*-cKO mice. The red arrows indicate the chromatoid body. Scale bars represent 2 micrometers.

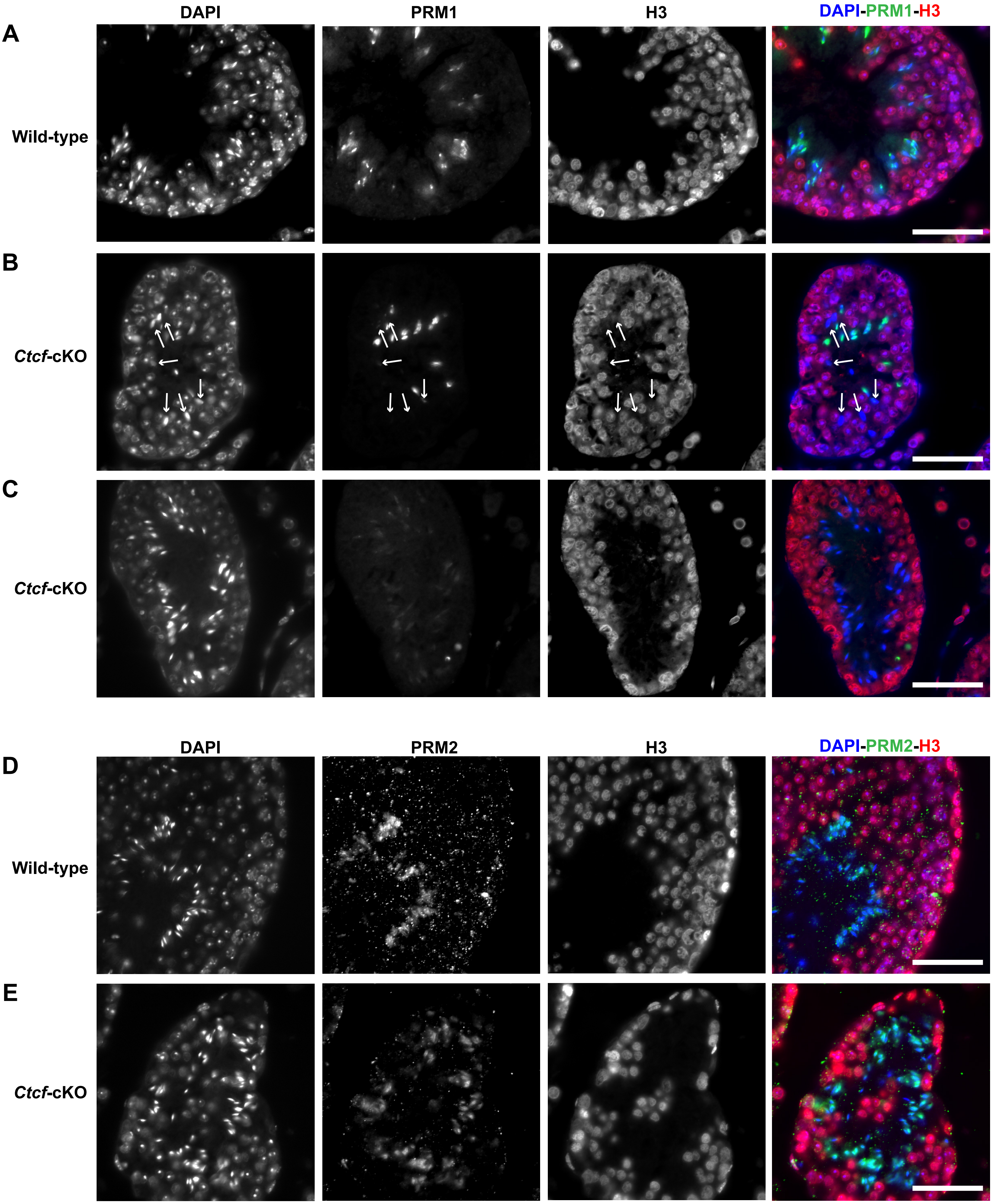
DAPI
PRM1
H3
DAPI-PRM1-H3
A
Wild-type
B
Ctcf-cKO
C
Ctcf-cKO
DAPI
PRM2
H3
DAPI-PRM2-H3
D
Wild-type
E
Ctcf-cKO

Supplementary Figure S4. PRM1 staining of elongated spermatids is drastically reduced in *Ctcf*-cKO testes.

(**A-C**) Labeling of wild-type and *Ctcf*-cKO testis sections by DAPI (detecting DNA) and antibodies against PRM1 and histone H3 (H3). Arrows in B indicate elongated spermatids that do not display a signal for PRM1, whereas in panel C the elongated spermatids display only a weak PRM1 staining signal. (**D-E**) Labeling of wild-type and *Ctcf*-cKO testis sections by DAPI (detecting DNA) and antibodies against PRM2 and histone H3 (H3). Scale bars in the merged panels represent 50 micrometers.

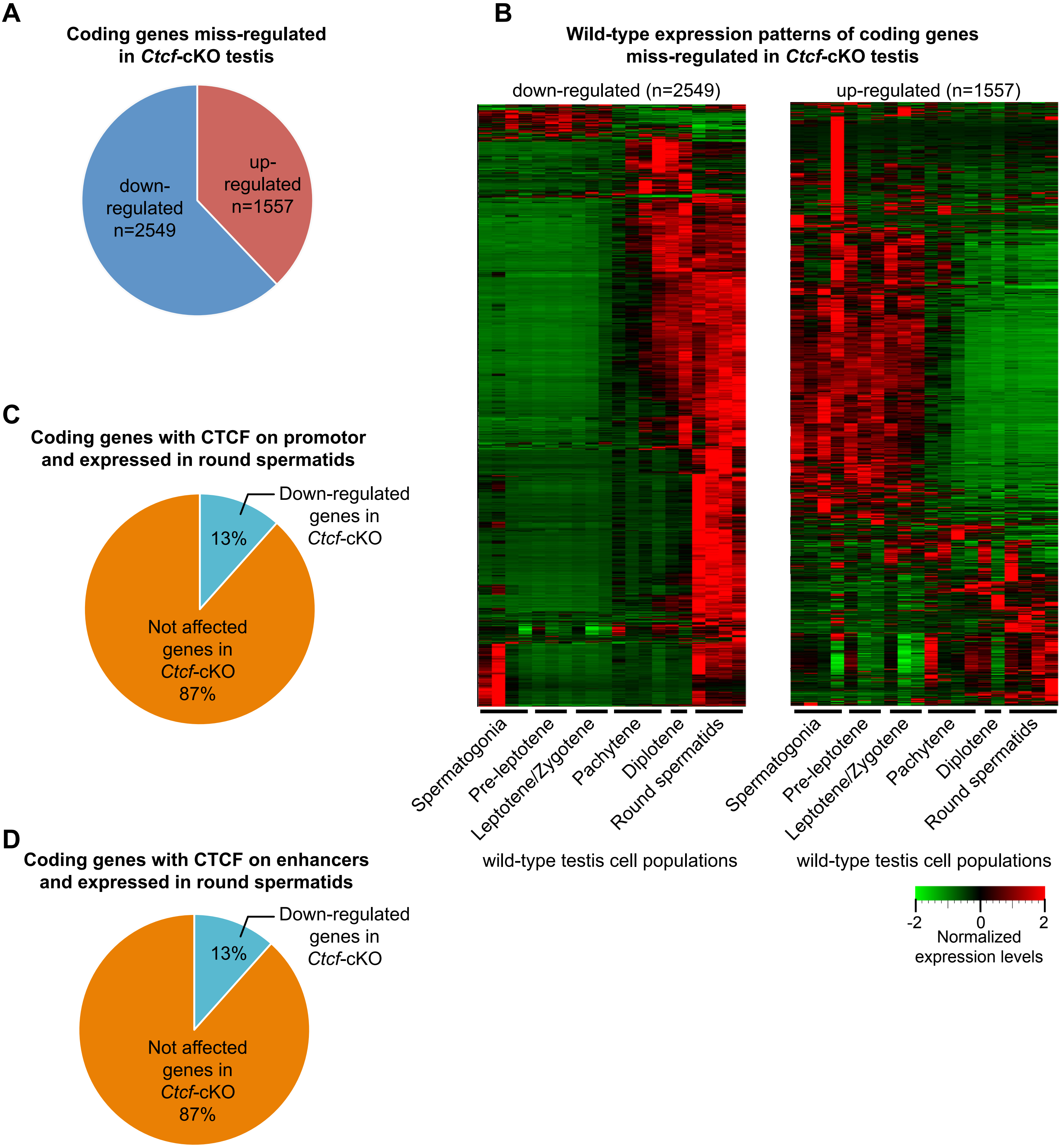
A
Coding genes miss-regulated in Ctcf-cKO testis
down-regulated n=2549
up-regulated n=1557
B
Wild-type expression patterns of coding genes miss-regulated in Ctcf-cKO testis
down-regulated (n=2549)
up-regulated (n=1557)
Spermatogonia
Pre-leptotene
Leptotene/Zygotene
Pachytene
Diplotene
Round spermatids
wild-type testis cell populations
Spermatogonia
Pre-leptotene
Leptotene/Zygotene
Pachytene
Diplotene
Round spermatids
wild-type testis cell populations
-2
0
2
Normalized expression levels
C
Coding genes with CTCF on promotor and expressed in round spermatids
Down-regulated genes in Ctcf-cKO
13%
Not affected genes in Ctcf-cKO 87%
D
Coding genes with CTCF on enhancers and expressed in round spermatids
Down-regulated genes in Ctcf-cKO
13%
Not affected genes in Ctcf-cKO 87%

Supplementary Figure S5. *Ctcf* deletion disrupts the expression of round spermatid genes

(**A**) 2549 coding genes down-regulated and 1557 coding genes up-regulated in *Ctcf*-cKO testis. (**B**) Analysis of the wild-type expression patterns of coding genes down-regulated (n=2549) and up-regulated (n=1557) in *Ctcf*-cKO testis. The wild-type expression patterns of the miss-regulated coding genes in *Ctcf*-cKO testis were retrieved from gene expression data from mouse staged cell populations and shown as heat maps. The normalized low versus high expression levels are represented on a green to red scale. (**C**) 169 of 1289 (13%) coding genes with CTCF on their promoter (plus/minus 2 Kb around TSS) and expressed in round spermatids of wild-type testis were down-regulated in *Ctcf*-cKO testis. (**D**) 376 of 2867 (13%) coding genes with CTCF on their enhancer (2-20 Kb upstream of the TSS) and expressed in round spermatids of wild-type testis were down-regulated in *Ctcf*-cKO testis.

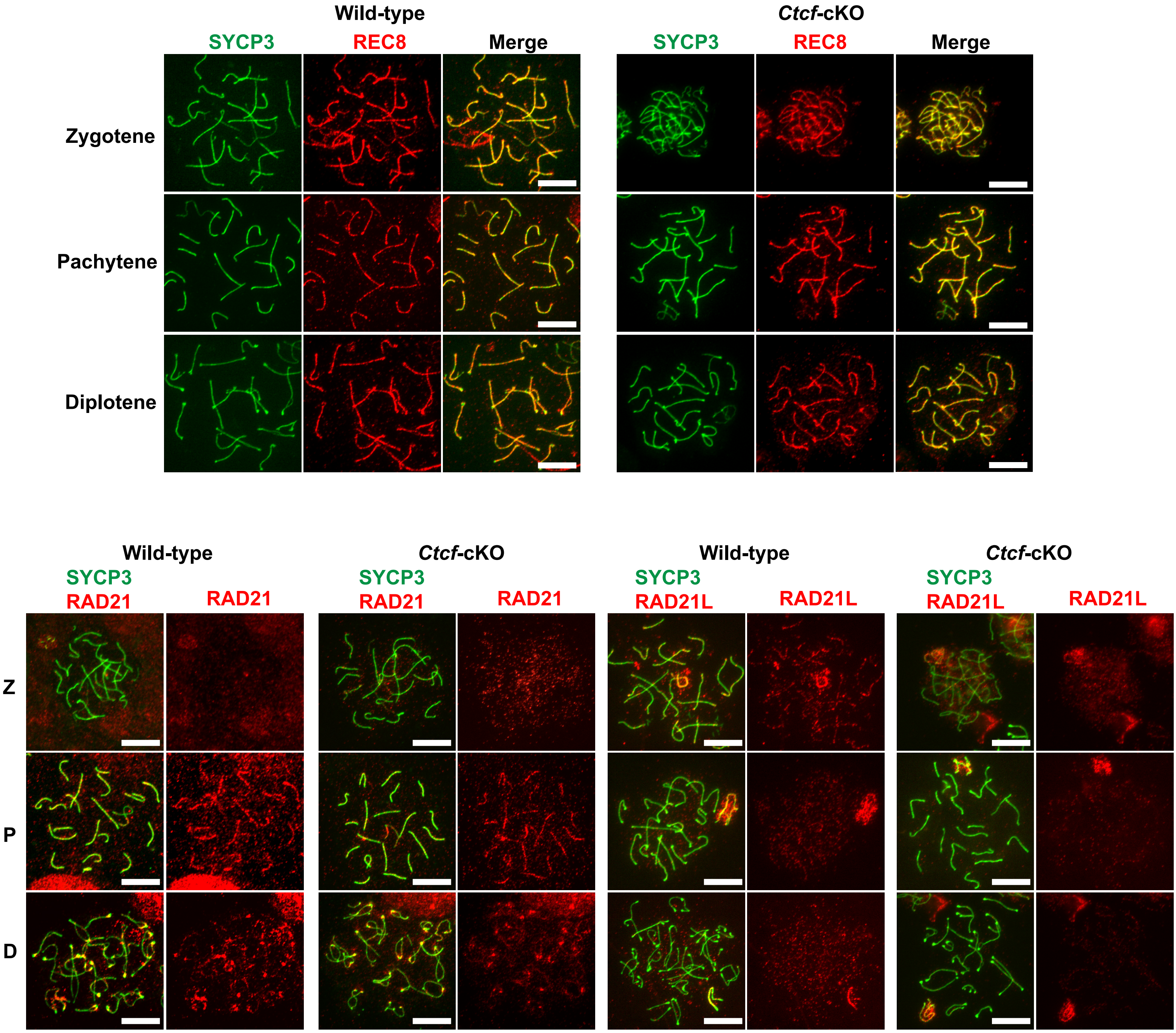
Wild-type
SYCP3
REC8
Merge
Ctcf-cKO
SYCP3
REC8
Merge
Zygotene
Pachytene
Diplotene
Wild-type
SYCP3
RAD21
RAD21
Ctcf-cKO
SYCP3
RAD21
RAD21
Wild-type
SYCP3
RAD21L
RAD21L
Ctcf-cKO
SYCP3
RAD21L
RAD21L
Z
P
D

Supplementary Figure S6. Localization of cohesin complex proteins in meiotic cells is not affected in *Ctcf*-cKO mice

The chromosomal localization of the cohesin complex proteins REC8, RAD21, and RAD21L in spermatocytes at the zygotene (Z), pachytene (P) and diplotene (D) stages of meiosis was not affected in *Ctcf*-cKO meiotic cells. DAPI staining was used to label chromatin and SYCP3 staining was used to provide staging of spermatocytes. Scale bars represent 10 micrometers.

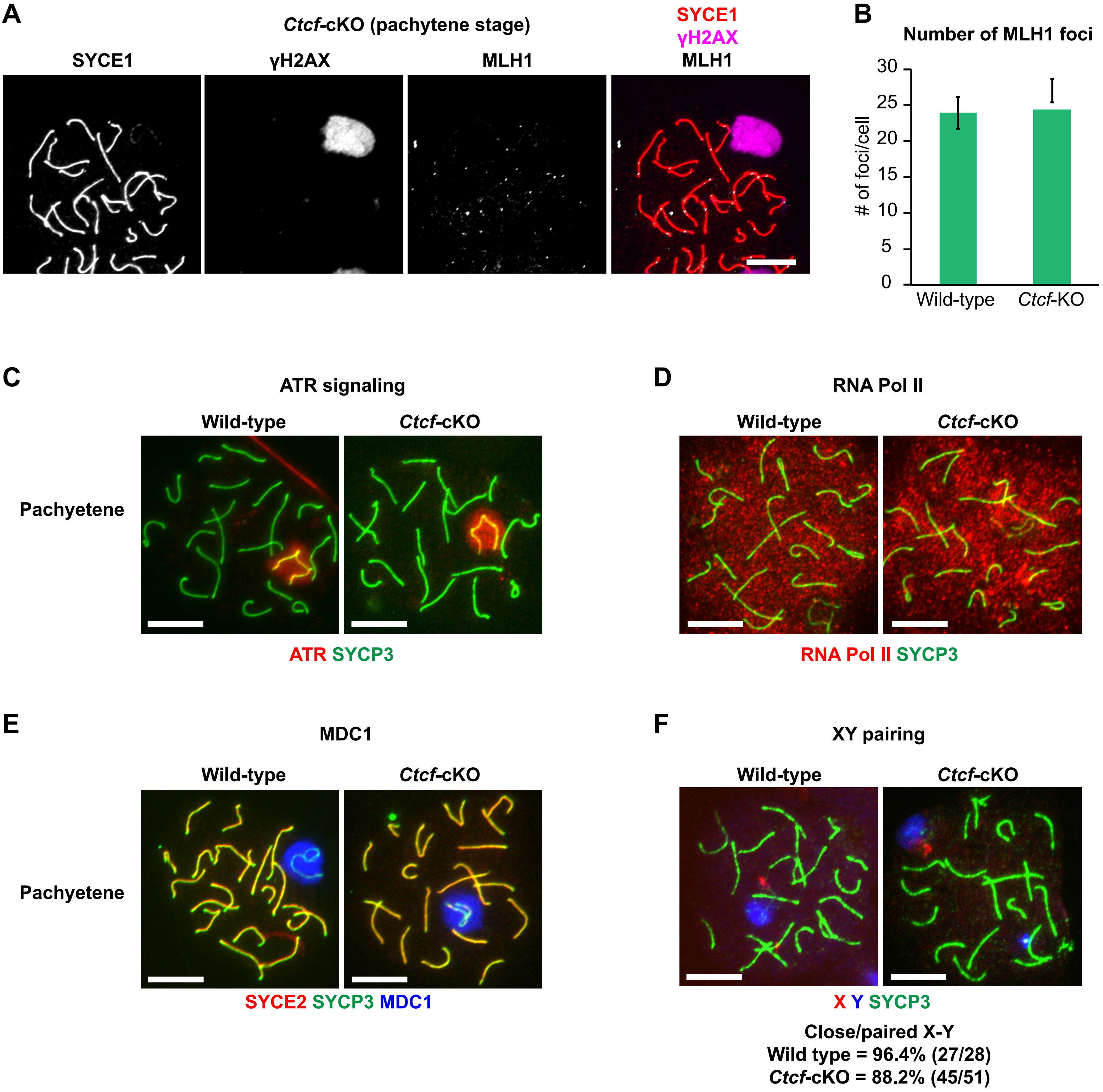
A
Ctcf-cKO (pachytene stage)
SYCE1
γH2AX
MLH1
SYCE1
γH2AX
MLH1
B
Number of MLH1 foci
# of foci/cell
30
25
20
15
10
5
0
Wild-type
Ctcf-KO
C
ATR signaling
Wild-type
Ctcf-cKO
Pachyetene
ATR SYCP3
D
RNA Pol II
Wild-type
Ctcf-cKO
RNA Pol II SYCP3
E
MDC1
Wild-type
Ctcf-cKO
Pachyetene
SYCE2 SYCP3 MDC1
F
XY pairing
Wild-type
Ctcf-cKO
X Y SYCP3
Close/paired X-Y
Wild type = 96.4% (27/28)
Ctcf-cKO = 88.2% (45/51)

Supplementary Figure S7. MLH1 localization to meiotic chromosomes and XY body formation are unperturbed in *Ctcf*-cKO mice

(**A**) MLH1 foci formation was analyzed in *Ctcf*-cKO pachytene cells. SYCE1 that labels fully synapsed meiotic chromosomes at the pachytene stage of meiosis, was used as a marker to identify pachytene cells. (**B**) Quantification of the numbers of MLH1 foci per cell (n=100) in wild-type and *Ctcf*-cKO pachytene cells. Three mice of each genotype were used. (**C-E**) Localization of XY body markers ATR (C), RNA Pol II (D), and MDC1 (E) was normal in *Ctcf*-cKO pachyetene spermatocytes. (**F**) Pairing of the XY body (as detected by chromosome painting) in pachytene spermatocytes, as scored by the presence of full SYCP3 axes, was not significantly altered in *Ctcf*-cKO cells as compared to the wild-type. Scale bars represent 10 micrometers.

Supplementary Data S1. Down-regulated coding genes in *Ctcf*-cKO testes

The gene list includes: gene symbol, description, chromosome of location, strand, start and stop sites and fold change (linear).

Supplementary Data S2. Up-regulated coding genes in *Ctcf*-cKO testes

The gene list includes: gene symbol, description, chromosome of location, strand, start and stop sites and fold change (linear).

Supplementary Data S3. Coding genes not affected in *Ctcf*-cKO testes

The gene list includes: gene symbol, description, chromosome of location, strand, start and stop sites and fold change (linear).

Supplementary Data S4. Functional annotation clusters of the down-regulated genes in the *Ctcf*-cKO testes, obtained by GO analysis with DAVID Bioinformatics Recourses website

The cluster with the highest enrichment score is highlighted with bold letters.

Supplementary Data S5. Down-regulated coding genes in *Ctcf*-cKO testes associated to sexual reproduction, spermatogenesis and sperm generation and grouped in the most enriched functional annotation cluster by the GO analysis in the DAVID Bioinformatics Recourses website

The genes known to affect spermiogenesis and display a similar phenotype as in this study are highlighted with red letters.

Supplementary Data S6. Functional annotation clusters of the up-regulated coding genes in *Ctcf*-cKO testes, obtained by GO analysis with DAVID Bioinformatics Recourses website

The three clusters with the highest enrichment score are highlighted with bold letters.

Supplementary Data S7. Coding genes with CTCF on promoter and enhancers, expressed in round spermatids and down-regulated in *Ctcf*-cKO testis
